# Arginine Side‐Chain Hydrogen Exchange: Quantifying Arginine Side‐Chain Interactions in Solution

**DOI:** 10.1002/cphc.201800598

**Published:** 2018-09-24

**Authors:** Harold W. Mackenzie, D. Flemming Hansen

**Affiliations:** ^1^ Institute of Structural and Molecular Biology Division of Biosciences University College London London WC1E 6BT United Kingdom

**Keywords:** ^13^C-detected NMR spectroscopy, arginine side-chains, guanidinium interactions, hydrogen exchange, protection factors, protein-protein interactions

## Abstract

The rate with which labile backbone hydrogen atoms in proteins exchange with the solvent has long been used to probe protein interactions in aqueous solutions. Arginine, an essential amino acid found in many interaction interfaces, is capable of an impressive range of interactions via its guanidinium group. The hydrogen exchange rate of the guanidinium hydrogens therefore becomes an important measure to quantify side‐chain interactions. Herein we present an NMR method to quantify the hydrogen exchange rates of arginine side‐chain ^1^H^ϵ^ protons and thus present a method to gauge the strength of arginine side‐chain interactions. The method employs ^13^C‐detection and the one‐bond deuterium isotope shift observed for ^15^N^ϵ^ to generate two exchanging species in ^1^H_2_O/^2^H_2_O mixtures. An application to the protein T4 Lysozyme is shown, where protection factors calculated from the obtained exchange rates correlate well with the interactions observed in the crystal structure. The methodology presented provides an important step towards characterising interactions of arginine side‐chains in enzymes, in phase separation, and in protein interaction interfaces in general.

## 1 Introduction

The backbone of proteins has long been the main focus of solution state Nuclear Magnetic Resonance (NMR) spectroscopy, where a comprehensive toolbox of elegant methods is now available to quantify structure,^1^, ^2^, ^3^, ^4^, ^5^ interactions,^6^,^7^ and dynamics over many order of magnitudes of timescales.^8^, ^9^, ^10^ An important method used to probe the formation of secondary structures and intramolecular interactions in proteins, such as hydrogen bonds, is the quantification of amide hydrogen exchange rates with the bulk solvent.^11^ These exchange rates are typically measured either by dissolving the protein of interest in ^2^H_2_O and following the decay of the amide proton signal‐intensities in NMR spectra^12^, ^13^, ^14^ or by magnetisation‐transfer type experiments.^15^, ^16^, ^17^, ^18^, ^19^ A comparison of the obtained hydrogen exchange rates obtained within proteins with the corresponding rates of small peptides yields a protection factor (PF),^20^ which reports on the energy of interactions formed by the amide proton in question.

Whilst a knowledge of protein backbone behaviour is often imperative to understand many aspects of protein functions, it is typically the side‐chains that directly mediate activity and participate in the important and functional interactions. Arginine residues are often crucial to many biological interaction interfaces because the flexible arginine side‐chain, consisting of a chain of aliphatic carbon atoms and a terminal guanidinium group, is capable of an array of hydrogen‐bonds and other interactions.^21^, ^22^, ^23^

The arginine guanidinium group has a very high p*K*_a_ of ∼14^24^ and the delocalised positive charge is therefore present under all physiologically relevant pH values.^25^ Each arginine guanidinium group has a large delocalised π‐system for cation‐π and π−π interactions^26^,^27^ as well as five guanidinium protons that are available for hydrogen‐bonding and salt‐bridging, but generally labile and able to exchange with the bulk solvent. The hydrogen exchange along with restricted rotation about the C^ζ^−N^ϵ^ bond are unfortunately often a hinderance to obtaining conventional ^1^H‐^15^N NMR correlation spectra at neutral or high‐pH.^28^,^29^ However, the rate of the hydrogen exchange can be dramatically reduced if the hydrogen in question is involved in a strong hydrogen‐bond or a salt‐bridge^30^,^31^ and a stronger interaction leads to a slower exchange rate. An accurate determination of the residue‐specific side‐chain hydrogen exchange rates therefore allows for a quantification of any such interactions involving an arginine side‐chain.

Below, we present a new NMR experiment to measure the hydrogen exchange rate of the ^1^H^ϵ^ proton of arginine side‐chains, based on ^13^C detection and the one‐bond deuterium isotope shift of ^15^N^ϵ^, which generates two exchanging species in ^1^H_2_O/^2^H_2_O mixtures.^17^ An application of the methodology to the 19 kDa protein T4 Lysozyme (T4 L) provides hydrogen exchange rates for 12 of the 13 arginine side‐chains in the protein. The remaining residue, R95, is characterised using an established method for monitoring hydrogen exchange. A subsequent comparison of the obtained exchange rates with those of free arginine allows for the calculation of side‐chain protection factors, which in turn report on the energetics of the interactions formed. Exchange rates between approximately 0.5−20 s^−1^ are accessible using the presented methods, however, as the hydrogen exchange rates are exquisitely sensitive to pH and temperature, small changes to the sample conditions allows measurements of rates in a large range of arginine side‐chains.

## 2 Results and Discussion

### 2.1 The NMR Experiment

**Figure 1:**
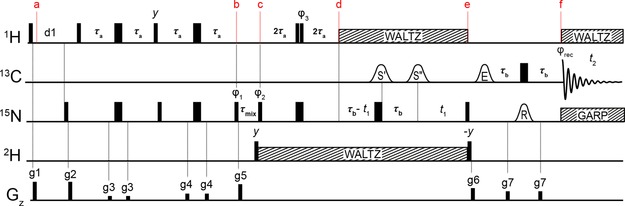
Pulse scheme of the new experiment to measure ^1^H^ϵ^/^2^H^ϵ^ hydrogen exchange rates in arginine side‐chains. The carrier positions are ^13^C: 156 ppm, ^15^N: 84 ppm, (78 ppm decoupling), ^1^H: 7 ppm and ^2^H: 7 ppm. Non‐selective 90° (180°) rf‐pulses are shown as narrow (wide) black bars and are applied at the highest available powers. The delay *τ*_a_ is 1/(4*J*_HN_)=2.72 ms and the delay *τ*_b_ is 1/(4*J*_CN_)=12.5 ms. The ^13^C frequency selective 180° pulses, S′ and S′′, are applied with a Seduce^32^ shape (300 μs at 18.8 T) and are selective for ^13^C^δ^ and ^13^C^ζ^ respectively. The ^13^C frequency selective 90° pulse, E, is applied with an EBURP‐2^33^ shape (1.5 ms at 18.8 T). The ^15^N 180° frequency selective pulse (R) is applied with a REBURP shape (3.75 ms at 18.8 T). Proton decoupling during the indirect chemical shift period and during acquisition is achieved with a 4 kHz WALTZ‐64^34^ scheme. ^15^N decoupling during acquisition is achieved with a 0.7 kHz GARP4^35^ scheme. ^2^H decoupling between *c* and *e* is achieved with a 1 kHz WALTZ‐16 scheme. Pulses are applied with *x* phase unless stated otherwise. The phase cycle used is ϕ_1_: *y*, −*y*, ϕ_2_: 2(*x*), 2(−*x*), ϕ_rec_: *x*, 2(−*x*), *x*. ϕ_3_ is cycled (*x*, −*x*) to implement the sign‐coding filter. Gradient pulses of 1 ms are represented by black rectangles and are applied with strengths of g1: 25.1 G/cm, g2: 25.1 G/cm, g3: 5.9 G/cm, g4: 9.1 G/cm, g5: 21.9 G/cm, g6: 16.6 G/cm, g7: 12.3 G/cm.

The NMR pulse scheme derived for the measurement of ^1^H^ϵ^/^2^H^ϵ^ exchange rates of arginine side‐chains is shown in Figure 1. The experiment incorporates several aspects of the previously published SOLEXSY^17^ method, as briefly described below. The sample is prepared in ^1^H_2_O/^2^H_2_O aqueous mixtures, for example 1 : 1 ^1^H_2_O : ^2^H_2_O although the exact ratio is not important. It is important to note that a $90_{x}^{o}$ ^1^H pulse followed by the gradient pulse g1 is inserted *before* the initial recovery delay to ensure that the amount of ^1^H^ϵ^ magnetisation present at the start of each transient is independent of the mixing time, *τ*_mix_. Following the recovery delay, equilibrium ^1^H^ϵ^ magnetisation is transferred to the protonated ^15^N^ϵ^ nuclei, ^15^N^ϵ^(^1^H), *via* the one‐bond ^1^H‐^15^N scalar coupling using a refocussed INEPT block between *a* and *b* in Figure 1. Since the deuterated ^15^N^ϵ^ species, ^15^N^ϵ^(^2^H), lacks a directly bound proton the refocussed INEPT results in a selective polarisation of only the ^15^N^ϵ^(^1^H) species, which leads to a density operator proportional to $N_{z}^{\epsilon }$ (^1^H) at point *b* using the product operator formalism^36^. During the variable mixing period between *b* and *c*, *τ*_mix_, magnetisation is partially transferred from $N_{z}^{\epsilon }$ (^1^H) to $N_{z}^{\epsilon }$ (^2^H) *via* the chemical exchange (hydrogen exchange) with the bulk solvent. Between points *c* and *d* a sign‐coding filter is present during which only the $N_{z}^{\epsilon }$ (^1^H) operator is inverted (ϕ_3_=*x*) or not inverted (ϕ_3_= ‐*x*) relative to the $N_{z}^{\epsilon }$ (^2^H) operator. Between *d* and *e* the chemical shift of ^15^N^ϵ^(^1^H/^2^H) is encoded in a semi‐constant time manner, whilst the one‐bond ^13^C^ζ^−^15^N^ϵ^ coupling is allowed to evolve for a total duration of 1/(2*J*_CN_)=25 ms, resulting in a density operator proportional to $2C_{z}^{\zeta }N_{z}^{\epsilon }$ (^1^H/^2^H) at point *e*. Frequency selective ^13^C^δ^ and ^13^C^ζ^ pulses are used to ensure that only the desired ^13^C^ζ^−^15^N^ϵ^ coupling evolves during this period. Simultaneous ^1^H and ^2^H WALTZ decoupling is applied throughout the indirect chemical shift period to suppress the scalar couplings associated with these nuclei. A final INEPT block between *e* and *f*, incorporating selective ^13^C^ζ^ and ^15^N^ϵ^ pulses refocuses the magnetisations of interest to in‐phase ^13^C^ζ^ at point *f* for detection under simultaneous ^1^H and ^15^N decoupling. Frequency discrimination in the indirect dimension is achieved by incrementing ϕ_2_ by 90° in line with the states‐TPPI scheme.^37^

**Figure 2:**
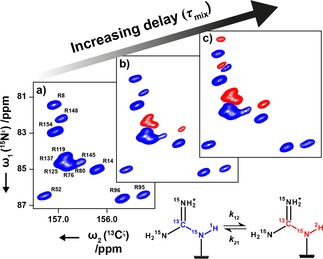
^13^C^ζ^‐^15^N^ϵ^ spectra of a sample of T4 L at pH 5.5 and 298 K recorded at 18.8 T. The mixing time, *τ*_mix_, was set to a) 1.5 ms, b) 50 ms and c) 300 ms. The increasing *τ*_mix_ allows the build‐up of ^15^N^ϵ^(^2^H). In each of the shown spectra ϕ_3_ was set to *x*, causing the inversion of the ^15^N^ϵ^(^1^H) signal (blue) with respect to the ^15^N^ϵ^(^2^H) signal (red).

The data are recorded as a pseudo‐4D experiment (ϕ_3_, *τ*_mix_, *t*_1_, *t*_2_), which after a two‐dimensional Fourier transformation along *t*_1_ and *t*_2_ results in two cross‐peaks, ^13^C^ζ^−^15^N^ϵ^(^1^H) and ^13^C^ζ^−^15^N^ϵ^(^2^H), for each arginine residue with the ^13^C^ζ^ frequency along the direct dimension (ω_2_) and the ^15^N^ϵ^ frequency along the indirect dimension (ω_1_). With *τ*_mix_ ∼0 s, only the ^13^C^ζ^−^15^N^ϵ^(^1^H) signals are observed (Figure 2a) and the spectrum resembles a standard ^13^C^ζ^−^15^N^ϵ^ HSQC experiment performed on a sample in ∼100 % H_2_O. Increasing the *τ*_mix_ delay causes initially the ^13^C^ζ^−^15^N^ϵ^(^2^H) cross‐peaks to appear for those residues undergoing the fastest exchange (Figure 2b). Increasing the delay further allows time for the more slowly exchanging signals to appear (Figure 2c). The intensity of both the ^15^N^ϵ^(^1^H) and ^15^N^ϵ^(^2^H) signals decay due to longitudinal relaxation during the mixing time, which limits the experiment to accurately probe hydrogen exchange rates faster than ∼0.5 s^‐1^. Furthermore, the experiment begins with a ^1^H−^15^N refocussed INEPT of ∼11 ms (Figure 1) and significant exchange during this period will lead to a reduction in the amount of $N_{z}^{\epsilon }$ (^1^H) magnetisation present at the start of *τ*_mix_, which limits the experiment to only probe hydrogen exchange rates slower than ∼20 s^−1^.

**Figure 3:**
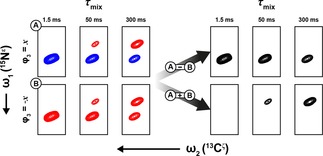
Generation of pseudo‐3D spectra reporting selectively on the intensity of the ^13^C^ζ^−^15^N^ϵ^(^1^H) and ^13^C^ζ^−^15^N^ϵ^(^2^H) cross‐peaks. The example shown is R14 in T4 L recorded at 18.8 T, pH 5.5, and a temperature of 298 K.

The ubiquitous large ^15^N one‐bond deuterium isotope shift (0.7±0.1 ppm in T4 L) makes assignment of the ^13^C^ζ^‐^15^N^ϵ^(^2^H) cross‐peaks trivial and ensures that the ^13^C^ζ^−^15^N^ϵ^(^1^H) and ^13^C^ζ^−^15^N^ϵ^(^2^H) cross‐peaks are well‐resolved. However, doubling the number of cross‐peaks in the spectrum increases the chance of overlap. In the ^13^C^ζ^−^15^N^ϵ^ spectrum of T4 L for example, R154 ^15^N^ϵ^(^2^H) partially overlaps with R148 ^15^N^ϵ^(^1^H), which hampers a quantitative analysis of either residue. To overcome this problem, a sign‐coding filter (Figure 1, *c* to *d*) is used immediately prior to the chemical shift evolution period to separate the ^15^N^ϵ^(^1^H) and ^15^N^ϵ^(^2^H) species into different sub‐spectra (Figure 3). Two experiments are recorded for each *τ*_mix_ delay with ϕ_3_=±*x*. The full pseudo‐4D dataset is split into two pseudo‐3D datasets after acquisition. The first dataset (ϕ_3_=+*x*) results in the ^15^N^ϵ^(^1^H) and ^15^N^ϵ^(^2^H) cross‐peaks having opposite sign, whilst in the second dataset (ϕ_3_=−*x*) both cross‐peaks have the same sign. Taking the difference of the two datasets thus provides a spectrum where only the protonated species, ^13^C^ζ^−^15^N^ϵ^(^1^H), are present. Similarly, addition of the two datasets provides the corresponding spectrum for the deuterated species, ^13^C^ζ^−^15^N^ϵ^(^2^H).

### 2.2 Deriving Hydrogen Exchange Rate Constants for T4 Lysozyme

**Figure 4:**
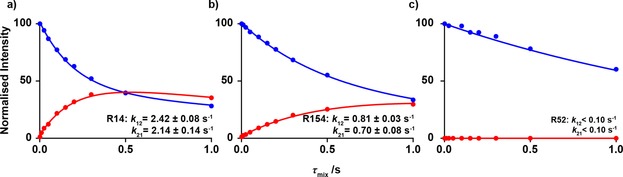
Intensities of ^13^C^ζ^−^15^N^ϵ^(^1^H) (blue) and ^13^C^ζ^−^15^N^ϵ^(^2^H) (red) as a function of *τ*_mix_ for three arginine side‐chains of T4 L. Circles represent experimental measurements, where experimental uncertainties are within the radius of the circles, and the line is a result of a least‐squares fit of Eq. (3) to the data shown. The rate constants *k*_12_ and *k*_21_ refer to the protium to deuterium and deuterium to protium exchange reactions, respectively. Data recorded on T4 L at 18.8 T, pH 5.5 and 298 K.

In Figures 2 and 3 it is seen that the line‐shapes are generally asymmetric, which is due to three‐bond ^15^N^ϵ^ and two‐bond ^13^C^ζ^ isotope shifts originating from ^1^H^η^/^2^H^η^. Peak volumes of ^13^C^ζ^−^15^N^ϵ^(^1^H) and ^13^C^ζ^−^15^N^ϵ^(^2^H) cross‐peaks as a function of *τ*_mix_ were therefore obtained by a simple summation over a region of approximately two linewidths in each dimension around the cross‐peak of interest. Examples of obtained build up/decay curves for R14, R154 and R54 of T4 L are shown in Figure 4, where hydrogen exchange rates are derived as described in the Experimental section.

The quality of the data is such that accurate exchange rate constants for both the protium to deuterium (*k*_12_) and deuterium to protium (*k*_21_) processes generally can be obtained. The ratio of these two rate constants is primarily dependent on the ratio of ^1^H_2_O and ^2^H_2_O present in the solution. For the data shown in Figure 4, where the amount of deuterium in the solvent was carefully maintained at 50 %, the ratio of the two processes (*k*_12_/*k*_21_) for R14 and R154 is ∼1.1, which reflects the fractionation factor previously reported for backbone amides.^38^,^39^

To avoid the need to very carefully control the relative amounts of protium and deuterium in the solvent, the total exchange constant is considered and defined to be the sum of the two contributing processes, *k*_ex_=*k*_12_+*k*_21_, which is independent of the protium/deuterium concentration. From the recorded data, accurate hydrogen exchange rate constants (*k*_ex_) can be obtained, 4.6±0.2 s^−1^ and 1.51±0.10 s^−1^ for R14 and R154, respectively. The extracted rates for the remaining residues are shown in Table 1(a). Five of the 13 arginine side‐chains in T4 L do not show any exchange in experiments performed at pH 5.5 and at 298 K with *τ*_mix_ up to 1 s. This is due to the fact that those hydrogens are engaged in interactions, such as a hydrogen‐bond with a carbonyl oxygen or salt‐bridges between the guanidinium group and acidic side‐chains, which is supported by the crystal structure of T4 L (PDB: 102L^40^). However, the data at pH 5.5 does not provide any quantitative information on the strength of the interactions.

As for the hydrogen exchange of backbone amide protons^13^ it is assumed that an arginine ^1^H^ϵ^ proton engaged in an interaction exchanges with the solvent via an “open state” and that the equilibrium between the *open* and *closed* states reports on the strength of the interaction formed. In the *closed* state, it is assumed that the exchange of the ^1^H^ϵ^ proton is negligible, while in the *open* state, exchange with the solvent proceeds unhindered as though the residue is ‘free’ [Eq. 1]:
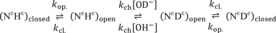



where *k*_op_ and *k*_cl_ are the first‐order rate constants for the reactions between the *open* and *closed* state, and *k*_ch_ is the second‐order rate constant for the base‐catalysed chemical hydrogen exchange.

A change in the sample conditions, which increases the exchange rate of free arginine therefore increases the exchange rate of interacting arginine side‐chains and ought to allow the observation of residues engaged in interactions. Exchange rate constants were therefore also obtained for T4 L at pH 7.4 and 308 K and these are shown in Table 1(b). Values for four of the five residues missing from measurements at pH 5.5 and 298 K are obtained at pH 7.4; only R95 continues to elude detection of exchange. It is interesting to note that the residues observed in the previous experiment (pH 5.5, 298 K) are not detected at pH 7.4, 308 K because the exchange rates are so fast that the magnetisation vanishes in the first refocussed INEPT. Overall, a combination of the two datasets here allows the measurement of hydrogen exchange rates for 12 of the 13 arginine residues in T4 L.

Only an upper bound for the hydrogen exchange rate of R95 can be estimated from the exchange experiment above since no visible exchange was observed within the mixing time used. To measure the hydrogen exchange rate of R95, T4 L was lyophilised from 100 % ^1^H_2_O at pH 5.5. The sample was subsequently dissolved in 100 % ^2^H_2_O before repeated ^1^H‐^15^N‐HSQC experiments were recorded at 278 K over the course of two days or at 288 K overnight (Supporting Information). The intensity of the ^1^H^ϵ^−^15^N^ϵ^ cross‐peak was measured as a function of time and fitted to a single exponential decay function. With these experiments the exchange rate of R95 ^1^H^ϵ^ can be estimated to 4.29±0.02×10^−5^ at 288 K, thus confirming that the side‐chain of R95 is engaged in a particularly strong interaction.

### 2.3 Comparison with Other Methods for Measurement of Hydrogen Exchange

One commonly used method to characterise hydrogen exchange is to dissolve the protein of interest in a ∼100 % ^2^H_2_O buffer and subsequently record ^1^H‐detected and ^15^N‐edited correlation spectra with regular intervals to quantify the time‐constant for the decay of the remaining ^1^H. The limitations of this commonly used experiment are two‐fold. Firstly, the dead time for dissolving the protein of interest in the ^2^H_2_O buffer and secondly the time required for each spectrum. On the other very extreme, very fast hydrogen exchange rates of up to 10^5^ s^−1^ can be measured using CPMG‐type experiments with and without proton decoupling^41^ and this approach has also been applied to arginine side‐chains. Thus, these two methods are both complementary to the method presented above.

A commonly used method to quantify hydrogen exchange on a timescale similar to the presented method is the CLEANEX‐PM^15^,^16^ experiment. In this experiment, the water ^1^H magnetisation is locked using a high‐power spin‐lock and the hydrogen exchange is quantified by observing an increase in the transferred proton magnetisation as a function of the time the water magnetisation is spin‐locked. There are several advantages of the presented method compared to the CLEANEX‐PM method. Firstly, in the method presented here the exchange takes place when the magnetisation is longitudinal and therefore there is no need to apply a long and strong spin‐lock field. The hydrogen exchange rate is very sensitive to temperature changes and the relatively high‐power field required to cover the full range of arginine ^1^H^ϵ^ chemical shifts can warm the sample significantly,^17^ affecting the measurements. Secondly, the CLEANEX‐PM experiment assumes the slow tumbling limit in order to achieve a cancelation of intramolecular NOE and ROE contributions. The effective correlation time of arginine side‐chains^28^ is often so that the slow‐tumbling limit is not applicable, as for the backbone of intrinsically disordered proteins.^17^ Thirdly, the use of ^13^C detection neatly sidesteps the need to suppress the solvent during acquisition, which can also affect cross‐peaks of interest. For example, in the ^1^H‐detected spectrum of T4 L, the ^1^H^ϵ^ resonance of R96 is just ∼1 ppm downfield from the H_2_O signal and can be affected by water suppression.

A recent and elegant method to quantify hydrogen exchange is the SOLEXSY method by Skrynnikov and co‐workers.^17^ As for the SOLEXSY method, the method presented in Figure 1 also employs the exchange between a protonated and a deuterated species to quantify hydrogen exchange and as such the two methods bear resemblance. However, the method presented here utilises ^13^C^ζ^ detection such that much fewer magnetisation transfer steps are required in the pulse scheme and magnetisation is kept on slower relaxing nuclei, such as ^13^C^ζ^ and ^15^N^ϵ^. Moreover, the arginine side‐chain does not contain a spin‐system that resembles the ^1^H^α^−^13^C^α^−^13^CO−^15^N network utilised in the SOLEXSY method, which means that the original SOLEXSY method is not directly applicable to quantify hydrogen exchange in arginine side‐chains.

It should be noted that there is an intrinsic sensitivity penalty in using the ^13^C^ζ^ nucleus for detection due to the lower gyromagnetic ratio when compared to ^1^H. However, we believe that the favourable relaxation properties of ^13^C^ζ^, the ability to encode a broad range of hydrogen exchange rates in relatively few 2D planes, and the inherent resistance to other transfer pathways clearly outweigh the intrinsic lower sensitivity associated with ^13^C detection.

### 2.4 Side‐Chain Protection Factors

**Figure 5:**
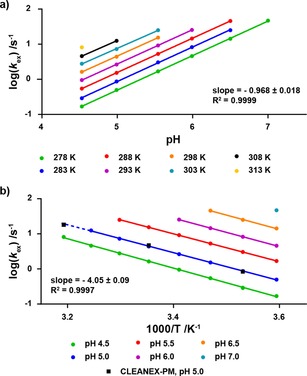
Plots demonstrating the linear dependence of log (*k*_ex_) on a) pH and b) inverse temperature. Data was recorded on [^13^C_6_,^15^N_4_]‐L‐arginine at 11.7 T. At high pH and high temperature, the exchange rate becomes too fast to measure. Black squares indicated data points measured using the CLEANEX‐PM experiment for comparison.

The method described here allows for a determination of the two pseudo first‐order rate constants, *k*_12_ and *k*_21_. In order to derive quantitative information about the strength of the interactions formed by the H^ϵ^ in question, a reference rate corresponding to a non‐interacting side‐chain H^ϵ^ needs to be available under the same experimental conditions. As seen above in Table 1, there is a strong dependence of the exchange rates on both pH and temperature, whereas little dependence on the ionic strength is expected^30^. As a reference exchange rate, hydrogen exchange data for free [^13^C_6_,^15^N_4_]‐L‐arginine was obtained over a large temperature and pH range (Figure 5). Specifically, the pH dependence of log (*k*_ex_) is linear with a slope of ∼1 as expected for a base‐catalysed reaction, and the temperature dependence follows an Eyring dependence (ΔH^≠^=78±2 kJ/mol). In turn, this means that reference hydrogen exchange rates for arginine H^ϵ^ can be calculated over a large range of pH and temperature. For example, using the linear dependences of log (*k*_ex_), the exchange rate of free arginine at pH 7.4 and 308 K was calculated to be 3100±100 s^−1^.

**Table 1:** Hydrogen exchange rates measured for arginine side‐chains in T4 L.

Residue	*k*_ex_ [s^−1^]^[a]^	*k*_ex_ [s^−1^]^[b]^	*PF*
R8	2.6±0.2	*ND* ^[d]^	6.0±0.5
R14	4.6±0.2	*ND* ^[d]^	3.4±0.2
R52	*ND* ^[c]^	8.9±1.8	348±70
R76^[e]^	4.24±0.16	*ND* ^[d]^	3.6±0.2
R80^[e]^	4.24±0.16	*ND* ^[d]^	3.6±0.2
R95	*ND* ^[c]^	*ND* ^[d]^	2.2±0.5×10^5[e]^
R96	*ND* ^[c]^	22±5	141±32
R119^[f]^	4.24±0.16	*ND* ^[d]^	3.6±0.2
R125^[f]^	4.24±0.16	*ND* ^[d]^	3.6±0.2
R137^[f]^	4.24±0.16	*ND* ^[d]^	3.6±0.2
R145	*ND* ^[c]^	1.4±0.4	2200±600
R148	*ND* ^[c]^	2.6±0.4	1200±200
R154	1.51±0.10	*ND* ^[d]^	10.2±0.8
Free Arg	15.5±0.5	3100±100^[g]^	

[a] Extracted rate constants, determined at pH 5.5, 298 K; [b] Exchange rate constants, determined at pH 7.4; 308 K; [c] Exchange too slow to be determined; [d] Exchange too fast to be determined; [e] Determined from ^1^H‐^15^N HSQC experiments as described in the Supporting Information; [f] Overlap of these residues prevented a residue‐specific measurement; [g] The rate for free arginine at pH 7.4 and 308 K is extrapolated from the linear dependence in Figure 5.

Hydrogen exchange rates were also measured for the methyl‐ester of [^13^C_6_,^15^N_4_]‐L‐arginine to confirm that possible intermolecular interactions between the guanidinium group and the carboxylic acid in free arginine did not affect the obtained exchange rates. Rapid hydrolysis of the methyl‐ester in the acidic aqueous conditions prevented a comprehensive analysis, but the results suggest that intermolecular interactions between the guanidinium group and the carboxylic acid, if present, are so weak that the hydrogen exchange rates are not affected.

**Figure 6:**
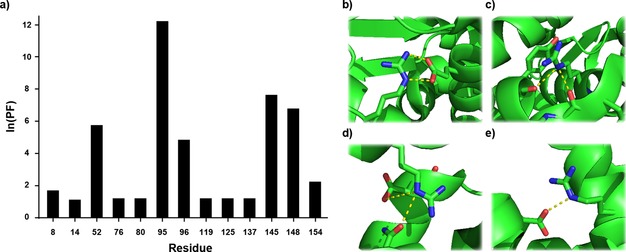
a) Quantification of the interactions formed by the arginine side‐chains in T4 L. Crystal structures of b) R52, c) R96, d) R145 and e) R148 obtained from PDB:102 L. Exchange data for R95 was obtained as described in the Supporting Information.

Once the hydrogen exchange rate for free arginine is determined it can be used as a reference to calculate a protection factor, PF, in line with protection factors for backbone amide groups, as [Eq. (2)]:^13^,^20^

(2) $$PF=\;\frac{k_{ex\left(free\right)}}{k_{ex}}\;$$

where *k*_ex(free)_ and *k*_ex_ are the hydrogen exchange rate constants obtained for free arginine and the arginine of interest, respectively. Calculated protection factors (*PF*) for the arginine side‐chains in T4 L are shown in Figure 6a and in Table 1. It is clear from the data that R52, R95, R96, R145 and R148 are all engaged in interactions that prevent the exchange of H^ϵ^ to varying degrees. This finding is in good agreement with the crystal structure (Figure 6b–e) as well as previous investigations into the rotational rate of the C^ζ^−N^ϵ^ bond.^22^

## 3 Conclusions

A new method for probing solution‐state interactions of arginine side‐chains in proteins has been presented. The presented ^13^C‐detected method allows measurements of the hydrogen exchange rate constant for the H^ϵ^ hydrogens of arginine side‐chains; when these rates are between ∼0.5 s^−1^ and 20 s^−1^. Thus, the method provides a means to quantify the interactions formed by the guanidinium group of arginine side‐chains in proteins. The dependence of the hydrogen exchange rate for free arginine on temperature and pH was determined over a large range to provide a reference from which arginine side‐chain protection factors can be calculated, similar to backbone amide protection factors that previously have been used widely to quantify interactions formed by the backbone in proteins. An application to the 19 kDa protein T4 Lysozyme was presented, which demonstrates the utility of the method and shows that for this protein protection factors for all bar one arginine side‐chain can be obtained. It should be stressed that arginine side‐chains that form salt‐bridges and medium‐strong hydrogen bonds are well captured under physiological conditions (pH 7.4, 308 K) using the presented method. Moreover, small changes in temperature and particularly in pH affect the rates substantially, thus generally allowing for a large range of arginine side‐chains to be characterised. It is envisaged that the new method serves as a particularly valuable tool to characterise active sites in enzymes,^21^,^26^ protein‐protein or protein‐nucleic acid interactions,^27^ and phase separation,^23^ where arginine residues are expected to play a crucial role for biological function.

## **Experimental Section**

Uniformly labelled [^13^C, ^15^N]‐T4 Lysozyme was overexpressed and purified from *Escherichia coli* as described previously.^42^ Following purification, the sample was exchanged into aqueous buffer (50 mM sodium phosphate, 25 mM NaCl, 2 mM EDTA, 2 mM NaN_3_, pH 5.5, 50 % ^2^H_2_O) and concentrated to ∼2 mM. The pH was measured using a Thermo Scientific Orion Star pH meter and is quoted uncorrected for the isotope effect.^43^ The pH was carefully raised to 7.4 with addition of small amounts of NaOH, prepared in 1 : 1 ^1^H_2_O:^2^H_2_O for subsequent experiments as required. Six samples of free [^13^C_6_,^15^N_4_]‐L‐arginine were prepared by dissolving an appropriate amount of [^13^C_6_, ^15^N_4_]‐L‐arginine hydrochloride in the above buffer to a final concentration of 50 mM. The pH of the samples were carefully adjusted to 4.5, 5.0, 5.5, 6.0, 6.5 and 7.0 with small additions of HCl or NaOH prepared in 1 : 1 ^1^H_2_O : ^2^H_2_O. The pH of each sample was measured immediately before and after each experiment.

[^13^C_6_,^15^N_4_]‐L‐arginine methyl ester
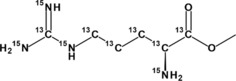



Anhydrous methanol (2.5 mL, 62 mmol) was added to a dried flask and cooled in an ice/water bath before acetyl chloride (0.65 mL, 9.1 mmol) was added dropwise under nitrogen. [^13^C_6_,^15^N_4_]‐L‐Arginine‐hydrochloride (102 mg, 0.462 mmol) was then added and the mixture allowed to warm to ambient temperature overnight. The solution was concentrated *in vacuo* and the residue lyophilised from H_2_O to give the desired product di‐hydrochloride salt as a white solid (120 mg, 0.443 mmol, 96 %). ^1^H NMR (400 MHz, ^2^H_2_O) δ 4.09 (d, *J*=147.3 Hz, 1H, Hα), 3.77 (d, *J*=3.9 Hz, 3H, CH3), 3.17 (d, *J*=139.9 Hz, 2H, Hδ), 2.27−1.28 (m, 4H, Hβ+Hγ). ^13^C NMR (101 MHz, ^2^H_2_O) δ 170.45 (d, *J*=61.8 Hz, C’), 156.71 (q, *J*=21.6 Hz, Cζ), 53.54 (s, CH3), 52.41 (ddt, *J*=61.8, 33.9, 6.0 Hz, Cα), 40.18 (ddd, *J*=35.8, 10.1, 5.3 Hz, Cδ), 26.88 (t, *J*=34.1 Hz, Cβ), 23.69 (t, *J*=35.2 Hz, Cγ). ESI‐MS (pos. *m/*z) 199.1 (100 % [M+H]^+^). TOFMS (pos. *m/z*) [M+H]^+^ calcd. for ^13^C_6_CH_17_^15^N_4_O_2_, 199.1434; found, 199.1434.

### NMR Spectroscopy

NMR experiments on T4 L were carried out at 298.2 K and 308.2 K on a Bruker Avance III (HD) spectrometer with a ^1^H operating frequency of 800 MHz equipped with a helium‐cooled TCI cryoprobe. NMR experiments on the samples of free arginine were carried out between 278.2 K and 313.2 K on a Bruker Avance III spectrometer with a ^1^H operating frequency of 500 MHz equipped with a nitrogen‐cooled prodigy cryoprobe and SampleJet sample changer. The pseudo‐4D experiments recorded on T4 L were acquired as 512×48 complex matrices with 10 *τ*_mix_ increments and sequential inversion of ϕ_3_ resulting in 20 planes. Spectral widths were 4000 Hz (^13^C), 800 Hz (^15^N) with carriers set to 156 ppm and 84 ppm respectively. The ^1^H and ^2^H carriers were set to 7 ppm. 80 scans were collected per *t*_1_ increment with *τ*_mix_ times of up 1 s resulting in a total experiment time of 64 hours. The experiments on free arginine were collected as above using 16 scans, a reduced spectral width with 16 complex points in ^15^N and *τ*_mix_ times of up to 3.5 s. The total experiment time was approximately 4.5 hours. CLEANEX‐PM experiments were recorded using the standard 1D sequence (*zgcxesgp*) provided in the Bruker pulse program library. 8192 complex points were recorded over 20 ppm with 16 scans and a recycle delay of 1 s. Selective excitation of H_2_O was achieved with a pulsed field gradient spin‐echo (PFG‐SE) element incorporating a 7.5 ms water‐selective Gaussian refocussing pulse. The CLEANEX spinlock field was applied at a strength of 4.8 kHz in the presence of a weak gradient of 0.1 Gcm^−1^ for durations of up to 50 ms.

### Data Analysis

NMR data was processed using NMRPipe^44^ and subsequently analysed using FuDA^45^. Peak volumes as a function of *τ*_mix_ were obtained by summation of the points in the spectrum within a radius of 0.25 ppm and 0.25 ppm in ^13^C and ^15^N dimension, respectively. Uncertainties, *σ*, in the obtained peak volumes were estimated by summation of an identical number of points in the region of the spectrum where no peaks were present.

The obtained peak volumes, *I*_1_(*τ*_mix_) and *I*_2_(*τ*_mix_), for the cross peaks reporting on the two species, ^15^N^ϵ^(^1^H) and ^15^N^ϵ^(^2^H) were subsequently used as input for least‐squares fitting analysis, where the model parameters, **p**, were extracted using in‐house written software (available upon request) by minimising the target function [Eq. (3)]:

(3) $$\chi^{2}\left(p\right)=\sum {\left(\frac{I_{1}^{exp}\left(\tau_{mix}\right)-I_{1}^{calc}\left(\tau_{mix}\right)}{\sigma }\right)}^{2}+{\left(\frac{I_{2}^{exp}\left(\tau_{mix}\right)-I_{2}^{calc}\left(\tau_{mix}\right)}{\sigma }\right)}^{2}$$

where the calculated intensities $I_{1}^{calc}\left(\tau_{mix}\right)$ and $I_{2}^{calc}\left(\tau_{mix}\right)$ are obtained by numerical integration of the Bloch‐McConnell equations [Eqs. (4), (4)]:^46^

(4) $$\frac{d}{d\tau }\left(\begin{array}{c} I_{1}^{calc}\left(\tau \right)\\ I_{2}^{calc}\left(\tau \right) \end{array}\right)=\left(\begin{array}{cc} -R_{1}^{N1H}-k_{12} & k_{21}\\ k_{12} & -R_{1}^{N2H}-k_{21} \end{array}\right)\left(\begin{array}{c} I_{1}^{calc}\left(\tau \right)\\ I_{2}^{calc}\left(\tau \right) \end{array}\right)$$

(5) $$\left(\begin{array}{c} I_{1}^{calc}\left(\tau \right)\\ I_{2}^{calc}\left(\tau \right) \end{array}\right)=\exp\left(\left(\begin{array}{cc} -R_{1}^{N2H}-k_{12} & k_{21}\\ k_{12} & -R_{1}^{N2H}-k_{21} \end{array}\right)\tau \right).\left(\begin{array}{c} I_{1}^{calc}\left(0\right)\\ I_{2}^{calc}\left(0\right) \end{array}\right)$$

The model parameters $R_{1}^{N1H}$ and $R_{1}^{N2H}$ are the longitudinal relaxation rate of the ^15^N^ϵ^(^1^H) and ^15^N^ϵ^(^2^H) species respectively, and *k*_12_ and *k*_21_ are the pseudo first‐order rate constants describing the reaction between ^15^N^ϵ^(^1^H) and ^15^N^ϵ^(^2^H). Furthermore, $I_{1}^{calc}\left(0\right)$ and $I_{2}^{calc}\left(0\right)$ are the initial intensities of the two species, where $I_{2}^{calc}\left(0\right)≃0$ by the design of the experiment. Hydrogen exchange during the sign‐coding filter was also taken into account, by propagating the Bloch‐McConnell equations over the 2τ_a_‐180^o^‐2τ_a_ element.

To obtain more stable fits, the ratio $R_{1}^{N2H}/R_{1}^{N1H}$ was fixed to 0.25 for free arginine and to 0.45 for arginine side‐chains within T4 L in the least‐squares optimisation. For an overall rotational correlation time $\tau_{C}=$ 10 ns and a local correlation time of $\tau_{e}=$ 500 ps, which mimics the scenario for T4 L, the calculated $R_{1}^{N2H}/R_{1}^{N1H}\;$ ratio is between 0.35 and 0.52 for order parameters, 0.2<*S* ^2^ <0.8. Importantly, changes to the assumed ratio have very minor effects on the obtained hydrogen exchange rates. For example, for the flexible R14 side‐chain the obtained exchange rate varied between 4.58 and 4.52 s^−1^ assuming ratios in the range from 0.15 to 0.45 and for the more rigid R52 side‐chain the obtained exchange rate varied between 9.05 and 8.87 s^−1^ for ratios in the range from 0.15 to 0.45.

## Conflict of interest

The authors declare no conflict of interest.

## Supporting information

As a service to our authors and readers, this journal provides supporting information supplied by the authors. Such materials are peer reviewed and may be re‐organized for online delivery, but are not copy‐edited or typeset. Technical support issues arising from supporting information (other than missing files) should be addressed to the authors.

Supplementary
