# Supplementary material for: Arginine Side‐Chain Hydrogen Exchange: Quantifying Arginine Side‐Chain Interactions in Solution
Source: Chemphyschem. 2018 Sep 24;20(2):252–9. doi: 10.1002/cphc.201800598 (PMC6391956; doi:10.1002/cphc.201800598)
Supplement: Supplementary file 1 — Supplementary [file CPHC-20-252-s001.pdf]

Supporting Information

© Copyright Wiley-VCH Verlag GmbH & Co. KGaA, 69451 Weinheim, 2019

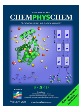

Arginine Side-Chain Hydrogen Exchange: Quantifying Arginine Side-Chain Interactions in Solution

Harold W. Mackenzie and D. Flemming Hansen*© 2018 The Authors. Published by Wiley-VCH Verlag GmbH & Co. KGaA.
This is an open access article under the terms of the Creative Commons Attribution License, which permits use, distribution and reproduction in any medium, provided the original work is properly cited.An invited contribution to a Special Issue on BioNMR Spectroscopy

1. Supplementary Tables

Table S1:

**Table S1.** Hydrogen exchange rates measured for [$^{13}C_6$,$^{15}N_4$]-L-arginine						
**Temp (K)**	**k_{ex} [s^{-1}]**					
	**pH 4.5**	**pH 5.0**	**pH 5.5**	**pH 6.0**	**pH 6.5**	**pH 7.0**
278.2	0.17 ± 0.02	0.50 ± 0.01	1.68 ± 0.02	4.58 ± 0.06	14.2 ± 0.3	46.9 ± 2.5
283.2	0.29 ± 0.01	0.87 ± 0.02	3.04 ± 0.03	8.25 ± 0.10	25.1 ± 0.8	
288.2	0.54 ± 0.01	1.53 ± 0.03	5.29 ± 0.05	14.5 ± 0.3	45.9 ± 3.3	
293.2	0.95 ± 0.02	2.64 ± 0.03	9.23 ± 0.15	25.5 ± 0.7		
298.2	1.64 ± 0.03	4.42 ± 0.08	15.5 ± 0.5			
303.2	2.78 ± 0.07	7.28 ± 0.30	25.0 ± 1.4			
308.2	4.58 ± 0.10	12.5 ± 0.6				
313.2	8.13 ± 0.36					

Table S2:

**Table S2.** Hydrogen exchange rate and protection factors for R95				
**Temp (K)**	**k_{ex} [s^{-1}]**		**PF/1000**	**ln(PF)**
	**Free arg.**	**R95**		
278.2	2.30	$8.36 \pm 0.04 \times 10^{-6}$	275	12.5
288.2	7.34	$4.29 \pm 0.02 \times 10^{-5}$	171	12.0
		**Average**	**223±52**	**12.3±0.2**

2. Supplementary Figure

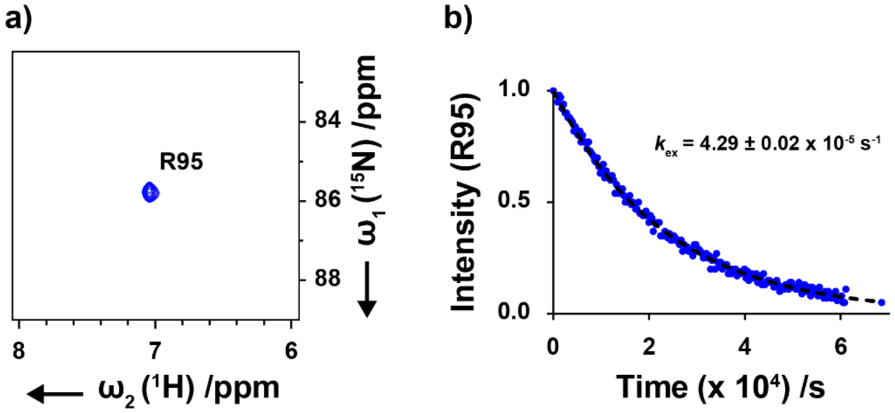

Figure S1. a) Arginine side-chain region of a ^{1}H-^{15}N HSQC spectrum of T4L just after dissolution into 100% $^{2}H_2O$ phosphate buffer recorded at 18.8 T at 288 K. The spectrum was acquired using an HSQC pulse sequence modified to be selective for arginine side chains (see experiment details) and clearly shows that R95 is exchanging much slower than the other 12 arginines in this protein. b) Plot of the intensity of R95 as a function of time, measured by repeated HSQC acquisitions over 16 hours. The intensity follows a single-exponential decay and a simple fitting procedure allows the rate constant (k_{ex}) to be extracted.

3. Pulse Programme

Arginine side-chain hydrogen exchange NMR experiment

```
; Filename: arg_hczne_hdx3d.2.hm
;
; Written on 1st March 2018 by Harold Mackenzie
;
; (H)CzNe HSQC for HDX measurement in arginine guanidinium groups
; 1Hepsilon excitation, 13Czeta detection
; pseudo-4D for measuring H/D exchange with sign-coding after mixing time
; semi-constant time in F1 (15Ne)
; Mixing times taken from vdlist
;
; F2[1He] > F3[15Ne] > t_mix [t2]> sign-coding [t3] > F3[15Ne, t1] > F1[13Cz, t4]
;
; WARNING: This sequence does not work with 'getprosol'. If you by accident
; type 'getprosol' using this sequence, then all parameters must be checked
; carefully.


;$CLASS=HighRes
;$DIM=4D
;$TYPE=
;$SUBTYPE=
;$COMMENT=

#include <Avance.incl>
#include <Delay.incl>
#include <Grad.incl>

;DEFINE PULSES

define pulse pwc
        "pwc=p1"                                        ;13C hard pulse at pl1
define pulse pwc_selr1
        "pwc_selr1=p11"                                 ;13C selective r-pulse at pl11
define pulse pwc_selr2
        "pwc_selr2=p12"                                 ;13C selective r-pulse at pl12
define pulse pwc_sele
        "pwc_sele=p13"                                  ;13C selective e-pulse at pl13

define pulse pwh
        "pwh=p2"                                        ;1H hard pulse at pl2

define pulse pwn
        "pwn=p3"                                        ;15N hard pulse at pl3
define pulse pwn_selr
        "pwn_selr=p31"                                  ;15N selective r-pulse at pl31

define pulse pwd
        "pwd=p4"                                        ;2H hard pulse at plw49

;DEFINE DELAYS

define delay taua
        "taua=1s/(cnst2*4)"                             ;1/4JHN
define delay taub
        "taub=1s/(cnst3*4)"                             ;1/4JCN

"d11= 30m"                                              ;Delay for disk
"d12= 2u"                                               ;Delay for power switching
"d16= 200u"                                             ;Delay for gradient recovery
```

```
"in0=inf1/2"                                       ;incremental delay
"d0=in0"

define list<delay> vd_list=<$VDLIST>               ;List for mixing time(s)
"l11=0"

;DEFINE OFFSETS

"spoffs11=0"                                       ;Czeta for seduce
"spoffs12=(cnst11*bf1)/1000000-o1"                 ;Cdelta for seduce
"spoffs13=0"                                       ;Czeta for eburp2
"spoffs31=0"                                       ;Nepsilon for reburp

;DEFINE ZERO POWER ON ALL CHANNELS USING SHAPED PULSES

"plw10=0"
"plw30=0"

;DEFINE CONSTANTS

"cnst31=o3/bf3"                                    ;Nepsilon
"cnst33=0.5*(cnst31+cnst32)"                       ;15N decoupling position

"cnst49=plw49"                                     ;2H decoupling power

;PULSE PROGRAM BEGINS

1 ze
  d11 LOCKDEC_ON                                   ;lock decoupling on
  50u LOCKH_ON                                     ;lock hold on
  d11 H2_PULSE                                     ;switch 2H to pulse
  d12 pl49:f4                                      ;power to decouple(2H)
2 d11 do:f2 do:f3 do:f4                            ;decoupling off

  ;purge 1H magnetisation before d1

  50u UNBLKGRAMP                                   ;gradient amp on
  d12 pl2:f2                                       ;power to high (1H)
  (pwh ph10):f2                                    ;90x
  2u
  p51:gp1                                          ;cleaning gradient
  d16
  50u BLKGRAMP                                     ;gradient amp off

  ;end 1H purge, start d1

  d11 H2_LOCK                                      ;switch 2H to lock
  d1*0.34                                          ;recycle delay
  6m LOCKH_OFF                                     ;lock hold off
  d1*0.66                                          ;recycle delay
  50u LOCKH_ON                                     ;lock hold on
  d12 H2_PULSE                                     ;switch 2H to pulse

  ;start purge equilibrium 15N magnetisation

  50u UNBLKGRAMP                                   ;gradient amp on
  d12 fq=cnst33(bf ppm):f3                         ;set 15N carrier
  d12 pl3:f3                                       ;power to high (15N)
  (pwn ph10):f3                                    ;90x
  2u
  p51:gp1                                          ;cleaning gradient
  d16
  d12 fq=cnst31(bf ppm):f3                         ;set 15N carrier, Ne

  ;end purge block

  ;start HN INEPT (Non-selective)
```

```
(pwh ph10):f2
"DELTA = taua-0.6366*pwh-2u-p52-d16-0.5*larger(pwh*2,pwn*2)"
DELTA
2u
p52:gp2
d16
( center (pwh*2 ph10):f2 (pwn*2 ph10):f3 )
2u
p52:gp2
d16
"DELTA = taua-0.5*larger(pwc*2,pwn*2)-2u-p52-d16-0.6366*pwh"
DELTA
(pwh ph11):f2
(pwn ph10):f3

;refocus 2HzNy to Nx

"DELTA = taua-0.6366*pwn-2u-p53-d16-0.5*larger(pwh*2,pwn*2)"
DELTA
2u
p53:gp3
d16
( center (pwh*2 ph10):f2 (pwn*2 ph10):f3 )
2u
p53:gp3
d16
"DELTA = taua-0.5*larger(pwc*2,pwn*2)-2u-p53-d16-0.6366*pwn"
DELTA
(pwn ph1):f3                                      ;Nx to Nz
2u
p54:gp4                                           ;cleaning gradient
d16
d12 pl10:f1                                       ;power to 0(13C)

;end INEPT, start mixing time for hydrogen exchange

"d2=vd_list[l11]"

if "d2<250m"
{
"DELTA=d2-2u-p54-d16-d12-pwd-d12"
DELTA                                             ;mixing time
}
else
{
"DELTA=d2-2u-p54-d16-d12-50u-d11-50m-6m-50u-d12-50u-pwd-d12"
50u BLKGRAMP                                      ;gradient amp off
d11 H2_LOCK                                       ;switch 2H to lock
50m
6m LOCKH_OFF                                      ;lock hold off
DELTA                                             ;mixing time
50u LOCKH_ON                                      ;lock hold on
d12 H2_PULSE                                      ;switch 2H to pulse
50u UNBLKGRAMP                                    ;gradient amp on
}

(pwd ph11):f4                                     ;2H flanking pulse (90y)
d12 cpd4:f4 ph10                                  ;2H decoupling on

(pwn ph2):f3                                      ;start sign-coding filter
"DELTA=2*taua-0.6366*pwn-larger(pwh,pwn)"
DELTA
(center (pwh ph10 pwh ph3):f2 (pwn*2 ph10):f3)
"DELTA=2*taua-larger(pwh,pwn)-d12-4u"
DELTA                                             ;end sign-coding
d12 pl29:f2
4u cpd2:f2 ph10
```

```
;evolve Ny to 2CzNx during semi-constant time chemical shift evolution

if "d0<(taub)"
{
"DELTA=taub-d0"
DELTA
(pwn*2 ph10):f3
(pwc_selr1:sp11 ph10):f1
"DELTA=taub-pwc_selr1-pwc_selr2"
DELTA
(pwc_selr2:sp12 ph10):f1
d0
}
else
{
(pwn*2 ph10):f3
"DELTA=d0-taub"
DELTA
(pwc_selr1:sp11 ph10):f1
"DELTA=taub-pwc_selr1-pwc_selr2"
DELTA
(pwc_selr2:sp12 ph10):f1
d0
}

(pwn ph10):f3                                   ;end t1

d12 do:f2 d12 do:f4                             ;1H, 2H decoupling off
(pwd ph13):f4                                   ;2H flanking pulse (90-y)
d12 pl30:f3                                     ;power to 0(15N)
2u
p55:gp5                                         ;cleaning gradient
d16
(pwc_sele:sp13 ph10):f1                         ;2CzNz to 2CyNz, Czeta eburp2

;start Ne selective block

"DELTA = taub-0.6366*pwc-2u-p56-d16-0.5*larger(pwc*2,pwn_selr)"
DELTA
2u
p56:gp6
d16 pl1:f1                                      ;power to high (13C)
( center (pwc*2 ph10):f1 (pwn_selr:sp31 ph10):f3 )
2u
p56:gp6
d16
30u fq=cnst33(bf ppm):f3                        ;set 15N carrier to decouple
d12 pl29:f2 pl39:f3                             ;power to decouple(1H,15N)
50u BLKGRAMP                                    ;gradient amp off
"DELTA = taub-0.5*larger(pwc,pwn_selr)-2u-p56-d16-30u-d12-50u"
DELTA

;end Ne selective block

;acquire Cx magnetisation with 15N, 1H decoupling

go=2 ph31 cpd2:f2 cpd3:f3
d11 do:f2 do:f3 mc #0 to 2
    F3QF(calph(ph3,+180))                       ;sign-coding
    F2QF(calclc(l11,1))                         ;t_mix
    F1PH(calph(ph2,+90), caldel(d0,+in0))       ;15Ne chemical shift

d11 H2_LOCK                                     ;switch H2 to lock
d11 LOCKH_OFF                                   ;lock hold off
d11 LOCKDEC_OFF                                 ;lock decoupling off

HaltAcqu, 1m
```

```
  stop, exit

;PHASE PROGRAMS

ph1=  1 3
ph2=  0 0 2 2
ph3=  0
ph31= 0 2 2 0

ph10= 0
ph11= 1
ph12= 2
ph13= 3

;DEFINITIONS

;pl1      : f1 channel - power level for hard pulse
;pl2      : f2 channel - power level for hard pulse
;pl3      : f3 channel - power level for hard pulse
;pl4      : f4 channel - power level for hard pulse
;pl29     : f2 channel - power level for CPD/BB decoupling
;pl39     : f3 channel - power level for CPD/BB decoupling
;pl49     : f3 channel - power level for CPD/BB decoupling
;sp11     : f1 channel - shaped pulse  180 degree (selective for Czeta)
;spnam11 : Seduce.100
;sp12     : f1 channel - shaped pulse  180 degree (selective for Cdelta)
;spnam12 : Seduce.100
;sp13     : f1 channel - shaped pulse  90 degree (selective for Czeta)
;spnam13 : Eburp2.1000
;sp31     : f3 channel - shaped pulse 180 degree (selective for Nepsilon)
;spnam31 : Reburp.1000
;p1       : f1 channel - 90 degree high power pulse
;p11      : f1 channel - 180 degree shaped pulse                [300 us at 18.8T]
;p12      : f1 channel - 180 degree shaped pulse                [300 us at 18.8T]
;p13      : f1 channel - 90 degree shaped pulse                 [1.5 ms at 18.8T]
;p2       : f2 channel - 90 degree high power pulse
;p3       : f3 channel - 90 degree high power pulse
;p31      : f3 channel - 180 degree shaped pulse               [3.75 ms at 18.8T]
;p4       : f4 channel - 90 degree high power pulse                      [250 us]
;p51      : homospoil pulse
;p52      : gradient pulse
;p53      : gradient pulse
;p54      : homospoil pulse
;p55      : homospoil pulse
;p56      : gradient pulse
;d0       : incremented delay
;d1       : relaxation delay
;taua     : 1/(4JHN)
;taub     : 1/(4JCN)
;d11      : delay for disk I/O                                            [30 ms]
;d12      : delay for power switching                                      [2 us]
;d16      : delay for homospoil/gradient recovery                        [200 us]
;cnst2    : J(HN)                                                         [92 Hz]
;cnst3    : J(CN)                                                         [20 Hz]
;cnst11   : Cdelta chemical shift offset                               [40.5 ppm]
;cnst31   : Nepsilon chemical shift offset                      [= o3p, 84.0 ppm]
;cnst32   : Neta chemical shift offset                                   [70 ppm]
;o1p      : Czeta chemical shift offset                                 [156 ppm]
;o2p      : Hepsilon/Heta chemical shift offset                           [7 ppm]
;o3p      : Nepsilon chemical shift offset                               [84 ppm]
;o4p      : Depsilon/Deta chemical shift offset                           [7 ppm]
;inf1     : 1/SW = 2 * DW
;in0      : 1/(2 * SW) = DW
;NS       : 4 * n
;DS       : 16
;td1      : number of experiments (15Ne chemical shift)
;td2      : number of experiments (mixing time points)                   [8-12]
;td3      : number of experiment (sign-coding filter)                       [2]
```

```
;FnMODE  : States-TPPI
;cpd2    : decoupling according to sequence defined by cpdprg2 (WALTZ64)
;pcpd2   : f2 channel - 90 degree pulse for decoupling sequence          [65 us]
;cpd3    : decoupling according to sequence defined by cpdprg3 (GARP4)
;pcpd3   : f3 channel - 90 degree pulse for decoupling sequence         [350 us]
;cpd4    : decoupling according to sequence defined by cpdprg3 (WALTZ16)
;pcpd4   : f4 channel - 90 degree pulse for decoupling sequence         [250 us]

;for z-only gradients:
;gpz1    : 47% (spoil)
;gpz2    : 11%
;gpz3    : 17%
;gpz4    : 41% (spoil)
;gpz5    : 31% (spoil)
;gpz6    : 23%

;use gradient files:
;gpnam1  : SMSQ10.100
;gpnam2  : SMSQ10.100
;gpnam3  : SMSQ10.100
;gpnam4  : SMSQ10.100
;gpnam5  : SMSQ10.100
;gpnam6  : SMSQ10.100
```

4. Supplementary Experimental Details

4.1 Quantification of the hydrogen exchange rate of H^{ε} of R95 in T4L

A 1 mM sample of U-[^{13}C,^{15}N]-T4L was lyophilised from a 50 mM sodium phosphate buffer solution at pH 5.5 containing 100% H_2O and subsequently dissolved in 100% ^2H_2O at 288 K. A series of $^1H^{\varepsilon}$-$^{15}N^{\varepsilon}$ HSQC experiments were recorded and the decay of the arginine side-chain cross-peak was monitored as a function of time. The pulse sequence used was based on the *hsqcetf3gp* code provided by Bruker, modified to use a 3.5 ms $^{15}N^{\varepsilon}$-selective REBURP[1] refocussing pulse in the initial INEPT transfer at a static field of 18.8 T. 500 μs gradient pulses of 6.96 Gcm^{-1} were applied either side of the selective pulse to minimise artefacts. The spectra were recorded as 1024 x 16 complex matrices with spectral widths of 16 ppm (^1H) and 10 ppm (^{15}N). The carriers are set to 4.7 ppm (^1H, on resonance with H_2O) and 84 ppm (^{15}N, REBURP pulse is applied at 75 ppm). 8 scans were collected per t_1 increment with a recycle delay of 1 s for a total experiment time of ~5 min. 200 spectra were recorded back-to-back for 16 hours after which the cross-peak intensity of R95 had decayed by over 90 %. The experiment was repeated at 278 K over 48 hours at 11.74 T to estimate uncertainties in the measurement.

5. Supplementary References

[1] H. Geen, R. Freeman, *J. Magn. Reson.* **1991**, *93*, 93–141.
